# Development of the Japanese version of Staff Attitude to Coercion Scale

**DOI:** 10.3389/fpsyt.2022.1026676

**Published:** 2022-10-17

**Authors:** Maiko Fukasawa, Michi Miyake, Takahiro Kikkawa, Tamio Sueyasu

**Affiliations:** ^1^Health Promotion Center, Fukushima Medical University, Fukushima, Japan; ^2^Department of Public Mental Health Research, National Institute of Mental Health, National Center of Neurology and Psychiatry, Kodaira, Japan; ^3^Faculty of Nursing, Undergraduate School of Medicine, Tokai University, Isehara, Japan; ^4^Department of Nursing, School of Health Sciences, Bukkyo University, Kyoto, Japan

**Keywords:** coercion, attitude, psychiatry, psychometric property, scale

## Abstract

**Background:**

An important factor in proceeding the efforts to reduce coercion in psychiatry is the attitudes of clinical staff toward its use. We aimed to develop the Japanese version of the Staff Attitude to Coercion Scale (SACS) and clarify its psychometric properties.

**Methods:**

After the translation and back-translation of the SACS, which includes 15 items consisting of three subscales, we conducted an anonymous self-administered questionnaire survey of clinical staffs working in 17 wards in two psychiatric hospitals. We administered the second survey to some of the participants to confirm the test-retest reliability. Additionally, we obtained information regarding the 17 wards from the institutions. Internal consistency was assessed using Cronbach’s alpha coefficients. Test-retest reliability was assessed using intraclass correlation coefficients (ICC). Structural validity was examined using confirmatory factor analysis (CFA) and exploratory factor analysis (EFA). For construct validity, the correlation of the SACS score within wards and its association with the actual use of seclusion/restraints were explored using multilevel multivariate linear regression analyses.

**Results:**

We used 261 (67.1%) responses, 35 responses of which were also used to examine test-retest reliability. Cronbach’s alpha coefficients (0.761) and ICC (0.738) indicated good reliability. The results of CFA based on the original three-dimensional structure did not indicate a good fit (CFA = 0.830, RMSEA = 0.088). EFA suggested a four-factor structure, two of which were almost consistent with the original two subscales. The correlation of the SACS score within wards was confirmed while a positive association with the actual use of seclusion/restraints was not identified.

**Conclusion:**

While the original three-dimensional structure was not replicated, construct validity was partially confirmed. Reliability of the total scale was good. In Japan, although using the subscales was not recommended, using the total scale of SACS seemed acceptable.

## Introduction

To protect the human rights of persons with disabilities (1), the use of coercion is an important theme in mental healthcare services. To reduce its use, various interventions have been implemented and several strategies have confirmed their efficacy (2–4).

To advance the efforts to reduce the use of coercion, the attitudes of clinical staff toward its use has been studied as an important domain (5–9). According to Doedens et al. (5), who conducted a systematic review of nursing staff attitudes toward coercive measures used in mental healthcare services, the attitudes toward coercion could be viewed from two paradigms: therapeutic and safety. In the therapeutic paradigm, the use of coercive measures is believed to positively affect patients as a treatment, while in the safety paradigm, it is considered undesirable but necessary to maintain the safety of patients and staff. Doedens et al. (5) revealed that, in the last two decades, the attitudes of nursing staff have shifted from the therapeutic to the safety paradigm.

Attitudes, perceptions, thoughts, or feelings influencing mental health nurses’ decision-making to use restraint have been identified (10). A previous study has demonstrated that staff’s attitudes are related to the tendency to use coercive measures in an experimental setting (11). Additionally, staff’s attitudes toward coercive measures have been reported to be influenced by training programs focusing on the use of coercive measures and the development of alternative skills (12, 13). It is important to assess the staffs’ attitudes toward coercion, not only because it affects their use of coercive measures directly, but also because it might affect the advancement of the various efforts in mental healthcare settings to reduce its use.

In Japan, as one of the scales to assess staff’s attitudes toward coercion, the Attitude to Containment Measures Questionnaire (ACMQ) (14) has been translated into Japanese (15). It assesses the staff’s attitudes in six dimensions toward 11 containment methods, each producing the degree of approval for each method. However, it has not been fully validated. Furthermore, it includes methods that are unfamiliar in Japan and is too complicated to use on site. To advance the efforts to reduce the use of coercion in Japan, a brief convenient scale with established reliability and validity is required to assess staff’s attitudes toward coercion.

One of the scales to measure clinical staff’s attitudes toward the use of coercion in psychiatric wards includes the Staff Attitude to Coercion Scale (SACS) (16). The SACS is a brief, self-administered questionnaire, developed in Norway, with good reliability and validity. It was developed through a series of procedures including item construction, pilot study, main study, and confirmation by experts. In the item construction process, three theories concerning the attitudes or reasons for using coercion or setting boundaries for patients were referred to, and the initial questionnaire containing 22 items was produced. In the pilot study, a three-dimensional structure was adopted, and the number of items was reduced to 15. Then, the three-dimensional structure of the 15-item questionnaire and its reliability were confirmed in the main study. In confirmatory principal component analyses, the three-dimensional model was replicated, which explained 49% of the variance. The internal consistency (Cronbach’s alpha coefficients) of the three subscales ranged from 0.69 to 0.73. Finally, construct validity was confirmed using another questionnaire survey for experts (16).

The SACS was developed in 2008 (16); it has been used worldwide and translated into several languages (17–26). According to a systematic review performed by Husum et al. (17), measurement properties of the SACS were reported in 13 studies conducted in Germany, Switzerland, Poland, the UK, the USA, India, Iran, Taiwan, and Norway, with six different language versions. Among them, structural validity was assessed in five studies and internal consistency was reported in 12 studies, the results of which suggested generally adequate validity and reliability of the translated scales. Making it available in Japan will not only advance the studies in Japan but also enable international comparison of the attitudes toward coercion, which would contribute to the deeper understanding of the use of coercion in the context of differences in national regulations and cultures.

The aim of this study, therefore, was to develop the Japanese version of the SACS and to clarify its psychometric properties among clinical staff working in psychiatric wards in Japan. Internal consistency, test-retest reliability, and structural validity were examined. Furthermore, for construct validity, the correlation within wards and the association to the actual use of seclusion/restraints in a ward were explored.

## Materials and methods

### Study design and participants

The questionnaire survey was conducted in two psychiatric hospitals in Japan between August and September, 2021. Both hospitals were located in urban areas, one in the North East area of Japan (Institution A) and the other in the capital region (Institution B). Institution A had approximately 280 beds with five psychiatric wards, including three ordinary wards, one acute ward, and one dementia ward. Institution B had approximately 640 beds with 12 psychiatric wards, including seven ordinary wards, three acute wards, and two recuperation wards. A self-administered anonymous questionnaire survey was conducted for all healthcare staff working in these 17 psychiatric wards. All the staff involved in the use of seclusion or restraints in psychiatric wards were invited to participate in the survey. Exclusion criteria were (1) those who had not been involved in administering seclusion or restraints, (2) those who did not work in a ward, and (3) those whose participation was deemed inappropriate by the head of the research. The exclusion criteria #3 had been set for unexpected problems arising; however, in the current survey, no one was excluded based on it. First, we explained the study aims, procedures, and voluntary nature of participation to the representative of the institutions and inquired the number of staff working in their psychiatric wards. Subsequently, we sent the questionnaires to the institutions and asked the institutions to distribute one to each of their staff. Attached to the questionnaire was an individual reply envelope and a document explaining our study aims, procedures, the voluntary nature of participation, anonymity, and protection of privacy. Informed consent was acquired by the participants sending back their questionnaire. As a recompense, we sent a 500 Japanese Yen cash voucher for the participants. We enclosed a postcard with the questionnaire and asked them to fill out their name and address separately to the questionnaire, so that the anonymity of the questionnaire was ensured. To obtain the information regarding the wards, we sent a questionnaire about the wards to the institutions and asked them to answer it for each ward.

To evaluate test-retest reliability, we asked the nurses in two wards to answer the questionnaire again 2 weeks after the initial survey. To link the two responses, we set a collaborator in these two wards in advance who produced a name list with identification numbers (ID). We asked them to distribute the questionnaire with ID based on the list in these two wards.

In this study, we reported measurement properties of the Japanese version of the SACS according to the terminology and definitions presented in the COnsensus-based Standards for the selection of health Measurement INstruments (COSMIN) study (27). The COSMIN Risk of Bias checklist (28–30) recommends that the sample size to explore structural validity using factor analysis be seven times the number of items and 100 or more. As the SACS includes 15 items, we intended to recover 105 or more responses in our survey. The expected response rate was 60% based on a similar questionnaire survey conducted recently, which targeted nurses working in a ward in Japan (31). Therefore, we planned to recruit at least 175 individuals.

### Variables

#### Staff Attitude to Coercion Scale

The SACS was developed in Norway by Husum et al. (16). It is a self-administered questionnaire including 15 items expressing the attitude toward the use of coercion to patients. It consists of three subscales: (I) coercion as offending (critical attitude); (II) coercion as care and security (pragmatic attitude); (III) coercion as treatment (positive attitude). The subscales include six, six, and three items, respectively. The items are assessed using a 5-point Likert scale ranging from 1 (disagree strongly) to 5 (agree strongly) and the means of the corresponding items are used. When using all the 15 items as a total scale, the items in subscale I (critical attitude) are reversed to represent a higher score indicating a more positive attitude toward the use of coercion. The Cronbach’s alpha coefficient for the total SACS has been reported as 0.78, and those for the subscale I, II, and III have been reported as 0.70, 0.73, and 0.69, respectively (16). The scale asks the representative attitudes toward the coercive measures within the ward or team in which the respondents are working, not the attitude of the respondents themselves.

We translated SACS into Japanese after obtaining permission from the original author (16). First, we made two Japanese versions: one was based on the English version attached to the original article (16) and the other was based on the original Norwegian version. The translation from the English version was conducted by one of the authors (MF) who is a native Japanese speaker and a researcher in the mental health field. The translation from the Norwegian version was conducted independently by a professional native Norwegian translator. We asked him/her for a literal translation. Next, based on these two Japanese versions and the English version, the three authors (MF, MM, and TK) developed a draft Japanese version by discussing it in a face-to-face meeting. We carefully considered whether each item reflects the literal and conceptual content of its English version and the expressions’ appropriateness in the Japanese context. We made inquiries to the original author to clarify the exact meaning of several original items by e-mail. In the process of developing the draft Japanese version, it was suggested that one tended to overlook the sentence requesting the participants to answer the questionnaire based on the representative attitude of the ward/team and tended to instead think about the attitude oneself. Therefore, we made this sentence bold and underlined. Subsequently, the draft Japanese version was back-translated into Norwegian by an independent native Norwegian speaker, who did not know about the original Norwegian version. The back-translated Norwegian version and the original Norwegian version were reviewed by the original author and their literal and conceptual equivalences were confirmed. Finally, we asked 10 Japanese nurses who had an experience of working in a psychiatric ward in Japan to answer the pre-final Japanese version of SACS and interviewed them on whether the items were understandable and subjectively relevant to the situations of Japanese psychiatric wards. We also asked them what they thought of the words “coercion/coercive measures” and “team.” For the pre-final Japanese version, amendments were not indicated.

#### Staff characteristics

The respondents’ demographic and work-related variables used in this study included age, sex, profession (doctor, nurse, assistant nurse, psychiatric social worker, psychologist, occupational therapist, pharmacist, or others), administrative or non-administrative position, employment status (full-time or part-time), years of experience in current profession, and experience of the member of the committee in minimizing confinement in an institution (the committee set-up in the psychiatric hospitals to monitor and review the use of seclusion and restraint and to organize staff training).

#### Ward characteristics

Ward-level variables used in this study included ward type, number of beds, number of seclusion rooms, number of nurses, mean number of inpatients per day, and total number and time of seclusion/restraints administered in a ward during 3 months (Supplementary Table 1). In Japan, the administration of seclusion and restraint in psychiatric hospitals is stipulated in the Notification of Japanese Health and Welfare Ministry, based on the Act on Mental Health and Welfare for the Mentally Disabled. When seclusion/restraints are implemented, the reason for its use and the start and end date must be recorded. To calculate the total time of seclusion/restraints by minutes during 3 months, we asked the institutions to transcribe the start and end times of all seclusion/restraints administered during the last 3 months (i.e., 1 May to 31 July 2021) from the ledgers of each ward. We inquired about all seclusion/restraints that occurred during the period, including those with a start time earlier than 1 May 2021. For the seclusion/restraints which did not end before 31 July 2021, we used 00:00 1 August 2021 as their end time. Concerning the total number of seclusion/restraints in a ward, we counted all of those administered in the ward during the 3 months, regardless of their start or end time. In addition, we asked the institutions to report the number of inpatients in each ward at the time of 00:00 every day during the 3 months, and averaged them to obtain the mean number of inpatients in each ward. To calculate the total time and number of seclusion/restraints in a ward per patients, we divided the total time/number of seclusion/restraints in a ward by the mean number of inpatients in each ward.

### Statistical analyses

First, for each item of SACS, we calculated the mean, standard deviation (SD), 95% confidence interval (95% CI), and minimum and maximum value to confirm the variability of the scores and its ceiling and floor effects. We then examined the reliability and validity of the Japanese version of SACS.

#### Reliability of the Japanese version of Staff Attitude to Coercion Scale

To evaluate reliability, the internal consistency and test-retest reliability were confirmed. For internal consistency, Cronbach’s alpha coefficients of the total score and each subscale were calculated. Cronbach’s alpha coefficients between 0.70 and 0.95 were considered sufficient (32). For test-retest reliability, ICCs were calculated for the total score, each subscale, and each item among the respondents who participated in the second survey and the initial survey. ICCs of at least 0.70 were considered acceptable (32).

#### Validity of the Japanese version of Staff Attitude to Coercion Scale

Structural validity was evaluated using confirmatory factor analysis (CFA) and exploratory factor analysis (EFA). CFA was performed to confirm the fit of our data to the original three-dimensional structure of SACS by Structural Equation Modeling using the full information maximum likelihood method. As indices of the level of fit, Chi-square statistic, Comparative Fit Index (CFI), and Root Mean Squared Error of Approximation (RMSEA) were used. CFI values equal to or above 0.95 were considered a good fit (33). RMSEA values less than or equal to 0.06 or 0.07 were considered a good fit, 0.08 or less were considered a reasonable fit, between 0.08 and 0.10 were considered a mediocre fit, and 0.10 or above were considered a poor fit (33). EFA was performed to explore the factor structure of our data. To check the adequacy of the sampling for factor analysis, we used Bartlett’s test for sphericity and calculated the Kaiser-Meyer-Olkin Measure of Sampling Adequacy (KMO-MSA). KMO-MSA in the 0.90 s indicates marvelous, 0.80 s indicates meritorious, 0.70 s indicates middling, and below 0.50 suggests unacceptable (34). The number of factors was determined by reference to Scree plot and Eigenvalues. For factor extraction, principal-components factor estimates with Promax rotation were used.

#### Hypotheses testing for construct validity

We confirmed the variance of the SACS score between wards and the association of the SACS score with the actual use of seclusion/restraints using multilevel linear regression analyses. Using the SACS score as an outcome, we developed a two-level multilevel multivariate linear regression model with individual SACS scores nested within wards. First, we hypothesized that the correlation of the SACS scores within wards is stronger than the correlation between wards, because of the respondents being required to answer based on the representative attitude in one’s ward/team. To confirm the variance across wards, we examined the ward level variance in a null model using the Likelihood Ratio test and calculated the ICC within wards. We additionally confirmed these indicators after adjusting for the respondents’ individual characteristics known to be related to their attitudes, such as age, sex, profession, and responsibility (24, 35). Subsequently, we examined the associations between the mean time and number of seclusion/restraints in a ward and the score of SACS after further adjustment of ward-level characteristics to test the hypothesis that increased use of seclusion/restraints in a ward is associated with a higher score of SACS.

All statistical analyses were performed using Stata 17.0 for Windows (StataCorp LP, College Station, TX, USA). Statistical significance was set at 0.05 and all tests were two-tailed.

### Ethical considerations

All procedures followed were in accordance with the ethical standards of the responsible committee on human experimentation, and with the 1964 Helsinki Declaration and its later amendments. The study protocol was reviewed and approved by the Ethics Committee of the National Center of Neurology and Psychiatry, Japan (A2021-047). For the institutions in which we conducted the survey, we explained the study aims, procedures, and voluntary nature of participation using a document and obtained written consent from the representatives of the institutions. For the individual staff members, we enclosed a document explaining the study aims, procedures, the voluntary nature of participation, anonymity, and protection of privacy with the questionnaire. Participants gave their consent by sending back their questionnaire.

## Results

### Respondent characteristics and scale descriptions

We sent 141 questionnaires to Institution A and 124 were returned (response rate 87.9%). We sent 248 questionnaires to Institution B and 146 (response rate 58.9%) were returned. In this study, we used 261 (67.1%) responses that did not have any missing information in the SACS. Respondents’ demographic and work-related characteristics are shown in Table 1. The mean age was 40.0 (SD = 11.2), ranging from 21 to 67. Nurses accounted for 77.0% of the participants and 11.1% were in an administrative position. The mean years of experience was 15.6 (SD = 10.7) and 23.8% had experienced the committee minimizing confinement.

**TABLE 1:** Demographic and work-related characteristics of the respondents (*N* = 261).

	Total		Institution A		Institution B		
				
			(*N* = 121)		(*N* = 140)		
					
	*N*	%	*N*	%	*N*	%	*p*
**Age**							
*Mean (SD)*	*40.0*	*11.2*	*40.6*	*11.5*	*39.4*	*11.0*	0.385
20–29	59	22.6	20	16.5	39	27.9	0.225
30–39	65	24.9	35	28.9	30	21.4	
40–49	80	30.7	37	30.6	43	30.7	
50–59	44	16.9	22	18.2	22	15.7	
60+	13	5.0	7	5.8	6	4.3	
**Sex**							
Men	118	45.2	58	47.9	60	42.9	0.411
Women	143	54.8	63	52.1	80	57.1	
**Profession**							
Doctor	15	5.8	4	3.3	11	7.9	0.002
Nurse	201	77.0	88	72.7	113	80.7	
Assistant nurse	8	3.1	7	5.8	1	0.7	
Psychiatric social worker	11	4.2	3	2.5	8	5.7	
Psychologist	3	1.2	3	2.5	0	0.0	
Occupational therapist	17	6.5	10	8.3	7	5.0	
Pharmacist	0	0.0	0	0.0	0	0.0	
Others	6	2.3	6	5.0	0	0.0	
**Administrative position**							
Yes	29	11.1	10	8.3	19	13.6	0.174
No	232	88.9	111	91.7	121	86.4	
**Employment status**							
Full-time	257	98.5	121	100.0	136	97.1	0.061
Part-time	4	1.5	0	0.0	4	2.9	
**Years of experience as the current profession^a^**							
*Mean (SD)*	*15.6*	*10.7*	*16.7*	*11.2*	*14.6*	*10.3*	0.120
<3 years	32	12.3	11	9.1	21	15.0	0.591
<6 years	29	11.1	13	10.7	16	11.4	
<10 years	26	10.0	11	9.1	15	10.7	
<20 years	79	30.3	38	31.4	41	29.3	
≥20 years	95	36.4	48	39.7	47	33.6	
**Experience of the member of the committee for minimizing confinement**							
Yes	62	23.8	16	13.2	46	32.9	<0.001
No	199	76.3	105	86.8	94	67.1	

^a^Including the experience other than psychiatric wards. The italicized values are the mean and SDs, while the other values are *N* and %.

Table 2 reports the minimum, maximum, means, SDs, and 95% CIs of each item of the SACS, as well as the total scale and original three subscales. The mean of the total scale was 3.08 (SD = 0.43), ranging from 1.80 to 4.47. The mean of each item ranged from 2.07 to 4.05. The distribution of each item score showed that the score of all the items ranged from 1 to 5 and the proportion of the highest and the lowest score for all items was below 30%. Ceiling or floor effects were not observed.

**TABLE 2:** Minimum, maximum, mean, standard deviation, and 95% confidence interval of the items of SACS calculated in the responses of the initial survey, and their intraclass correlation coefficient calculated among those who participated both in the initial and the second survey (*N* = 261).

		Min	Max	Mean^a^	SD	95% CI		Test-retest reliability (*N* = 35)			
							
								ICC	95 % CI		*p*
**I. Coercion as offending subscale**											
3	Use of coercion can harm the therapeutic relationship	1.00	5.00	3.75	0.90	3.64	3.86	0.600	0.331	0.778	<0.001
4	Use of coercion is a declaration of failure on the part of the mental health services	1.00	5.00	2.20	0.87	2.09	2.30	0.439	0.123	0.673	0.004
8	Coercion violates the patients integrity	1.00	5.00	3.70	1.00	3.58	3.82	0.512	0.216	0.721	0.001
13	Too much coercion is used in treatment	1.00	5.00	2.44	0.90	2.33	2.55	0.370	0.042	0.625	0.014
14	Scarce resources lead to more use of coercion	1.00	5.00	3.42	1.04	3.29	3.54	0.464	0.168	0.686	0.001
15	Coercion could have been much reduced, giving more time and personal contact	1.00	5.00	3.46	0.98	3.34	3.58	0.371	0.043	0.626	0.014
	**Mean**	**1.50**	**5.00**	**3.16**	**0.54**	**3.10**	**3.23**	**0.563**	**0.293**	**0.751**	<0.001
**II. Coercion as care and security subscale**											
1	Use of coercion is necessary as protection in dangerous situations	1.00	5.00	4.02	0.69	3.93	4.10	0.666	0.435	0.815	<0.001
2	For security reasons coercion must sometimes be used	1.00	5.00	4.05	0.68	3.96	4.13	0.735	0.534	0.857	<0.001
5	Coercion may represent care and protection	1.00	5.00	3.57	0.88	3.47	3.68	0.625	0.376	0.790	<0.001
7	Coercion may prevent the development of a dangerous situation	1.00	5.00	3.67	0.84	3.56	3.77	0.599	0.334	0.776	<0.001
9	For severely ill patients coercion may represent safety	1.00	5.00	3.62	0.89	3.51	3.73	0.550	0.273	0.744	<0.001
11	Use of coercion is necessary toward dangerous and aggressive patients	1.00	5.00	3.44	0.91	3.33	3.56	0.572	0.301	0.758	<0.001
	**Mean**	**1.00**	**5.00**	**3.73**	**0.58**	**3.66**	**3.80**	**0.832**	**0.691**	**0.912**	<0.001
**III Coercion as treatment subscale**											
6	More coercion should be used in treatment	1.00	5.00	2.07	0.87	1.96	2.18	0.275	−0.068	0.557	0.056
10	Patients without insight require use of coercion	1.00	5.00	2.42	0.96	2.30	2.54	0.642	0.401	0.801	<0.001
12	Regressive patients require use of coercion	1.00	5.00	2.29	0.83	2.19	2.39	0.615	0.364	0.784	<0.001
	Mean	**1.00**	**5.00**	**2.26**	**0.71**	**2.17**	**2.35**	**0.646**	**0.407**	**0.803**	<0.001
**Total of the 15 items^b^**		**1.80**	**4.47**	**3.08**	**0.43**	**3.03**	**3.13**	**0.738**	**0.540**	**0.859**	<0.001

^a^Score range: 1 (disagree strongly) to 5 (agree strongly). ^b^Scores in the subscale I were reversed. The bold values are the summary scores of each subscale.

### Reliability of the Japanese version of Staff Attitude to Coercion Scale

Regarding the internal consistency, the Cronbach’s alpha coefficient of all the 15 items was 0.761. Cronbach’s alpha coefficients for Subscales I, II, and III were 0.570, 0.804, and 0.723, respectively.

To confirm the test-retest reliability, 44 participants were asked to answer SACS twice, and 37 returned both the initial and the second questionnaire, among which we used 35 responses of which there was no missing information in both SACS. The mean period between the two responses was 27.5 days (SD = 8.0). The ICC of each item, as well as the total scale and three subscales, are reported in Table 2. The ICC of the total scale was 0.738 (95% CI: 0.540–0.859), and that of each item ranged from 0.275 to 0.735.

### Validity of the Japanese version of Staff Attitude to Coercion Scale

**FIGURE 1:**
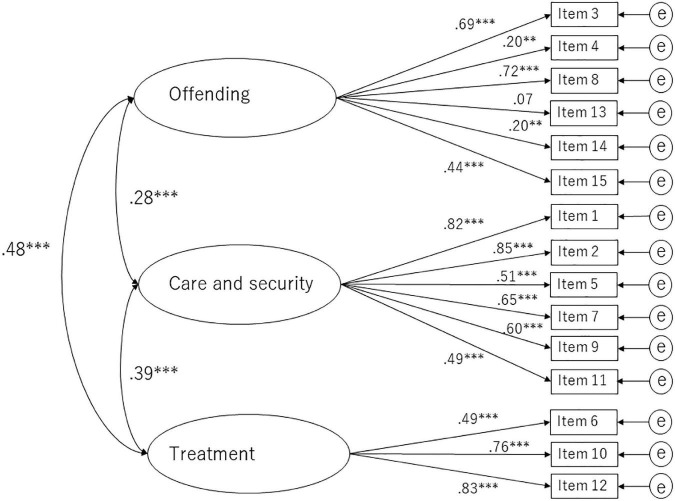
Confirmatory factor analysis based on the original three-dimensional structure of SACS. ^**^p < 0.01; ^***^p < 0.001.

For structural validity, we conducted CFA based on the original three-dimensional structure (Figure 1). Scores in subscale I (offending) were reversed. The path from the latent factor named offending to item 13 was not significant and those to items 4 and 14 were weak. The goodness-of-fit indices were as follows: Chi-square = 263.267 (degree of freedom = 105, *p* < 0.001); CFI = 0.830; RMSEA = 0.088 (95% CI: 0.076–0.101), which indicated a marginally acceptable but not a good fit.

The adequacy of the data for EFA was confirmed by Bartlett’s test for sphericity (Chi-square = 1109.774, degree of freedom = 105, *p* < 0.001) and KMO-MSA (0.782). From the EFA of our data, four factors were extracted (Table 3). The Scree plot showed four factors with an Eigenvalue higher than 1. The curve of the plot and each factor’s interpretability also suggested four factors. These four factors explained 58.49% of the variance. Factors 1 and 2 corresponded to the original subscale II (care and security) and III (treatment), respectively, except for item 11. Item 11 was originally included in the subscale II (care and security), while in our data, it loaded most heavily on Factor 2 (treatment) and also had a factor loading greater than 0.4 for Factor 1 (care and security). Items 5 and 6 also loaded on Factor 3 with factor loadings greater than 0.4. The items in the original subscale I (offending) loaded on Factors 3 and 4, and item 4 did not load on any factors sufficiently. Factor 3 seemed to indicate the use of coercion is offending, while Factor 4 seemed to indicate that the use of coercion can be decreased or is avoidable.

**TABLE 3:** Factor loadings of SACS items based on exploratory factor analysis (*N* = 261).

		Factor 1	Factor 2	Factor 3	Factor 4
**I. Coercion as offending subscale^a^**					
3	Use of coercion can harm the therapeutic relationship	−0.058	0.125	**0.706**	0.044
4	Use of coercion is a declaration of failure on the part of the mental health services	**0.381**	−0.316	0.266	0.297
8	Coercion violates the patients integrity	0.007	0.050	**0.811**	0.013
13	Too much coercion is used in treatment	0.198	−0.228	−0.070	**0.609**
14	Scarce resources lead to more use of coercion	−0.234	0.041	−0.021	**0.816**
15	Coercion could have been much reduced, giving more time and personal contact	−0.074	0.334	0.198	**0.585**
**II. Coercion as care and security subscale**					
1	Use of coercion is necessary as protection in dangerous situations	**0.838**	0.024	−0.052	−0.002
2	For security reasons coercion must sometimes be used	**0.829**	0.006	0.062	−0.058
5	Coercion may represent care and protection	**0.545**	−0.108	0.454	−0.131
7	Coercion may prevent the development of a dangerous situation	**0.737**	0.147	−0.108	−0.127
9	For severely ill patients coercion may represent safety	**0.625**	0.260	0.018	−0.043
11	Use of coercion is necessary toward dangerous and aggressive patients	0.439	**0.562**	−0.260	0.186
**III. Coercion as treatment subscale**					
6	More coercion should be used in treatment	−0.142	**0.475**	0.410	−0.053
10	Patients without insight require use of coercion	0.074	**0.789**	0.054	0.002
12	Regressive patients require use of coercion	0.099	**0.753**	0.135	−0.023

^a^Scores of the items in subscale I were reversed. The bold values are the summary scores of each subscale.

### Hypotheses testing for construct validity

Tables 4A,B report the results of the multilevel multivariate linear regression analyses using the SACS score as a dependent variable, considering the within-ward correlation. The SACS score was the mean of the 15 items, each of which ranged from 1 to 5. We used the data from 255 respondents who answered regarding the ward in which they belong or mainly work in. The number of respondents per ward ranged from 7 to 29. In the null model, ward level variance was 0.01, its standard error (SE) was 0.01, and its 95% CI was 0.00–0.03, indicating a small but significant variance (Table 4A). The Likelihood Ratio test indicated a significant improvement of the model by adding the ward level variance (*p* = 0.022). The ICC was 0.04. After adjusting for the individual characteristics (Model 1), ward level variance was 0.01 (SE = 0.01, 95% CI = 0.00–0.04) and the ICC was 0.06. The Likelihood Ratio test still indicated the significant improvement of the model by adding the ward level variance (*p* = 0.007). In Models 2 and 3, we examined the associations of the actual use of seclusion/restraints in a ward with the SACS score (Table 4B). The total time of both the seclusions and restraints in a ward per patient was not significantly associated with the SACS score (Model 2-1, 2-2). On the other hand, while the total number of seclusions was not (Model 3-1), total number of restraints was significantly and negatively associated with the SACS score (Model 3-2). Regarding the individual characteristics, we did not adjust for the respondents’ age due to its high correlation with their years of experience. Using age instead of the years of experience did not change the results (results not shown).

**TABLE 4A:** Ward level variance of the SACS score using multilevel linear regression analyses (*N* = 255, 17 wards).

	Null model				Model 1			
		
	Coef.	SE	*p*		Coef.	SE	*p*	
Intercept	3.09	0.03	<0.001		3.21	0.22	<0.001	
**Individual characteristics**								
Sex (women)					−0.02	0.05	0.740	
**Professions (ref. nurse)**								
Doctor					0.31	0.13	0.020	
Occupational therapist					0.20	0.10	0.058	
Psychiatric social worker					−0.34	0.12	0.006	
Others					0.00	0.11	0.993	
Years of experience					0.00	0.00	0.581	
Administrative position					0.00	0.10	0.998	
Employment status (full-time)					−0.10	0.21	0.633	
Experience of the committee for minimizing confinement					−0.02	0.06	0.739	

	**Estimate**	**SE**	**95% CI**		**Estimate**	**SE**	**95% CI**	

Ward level variance/SE/95% CI	0.01	0.01	0.00	0.03	0.01	0.01	0.00	0.04
Individual level variance/SE/95% CI	0.17	0.02	0.14	0.20	0.15	0.01	0.13	0.18
Intraclass correlation coefficient (ICC)	0.04				0.06			

Coef., Coefficient; SE, standard error; ref., reference; 95% CI, 95% confidence interval.

**TABLE 4B:** Associations between the SACS score and duration and frequency of seclusion/restraints administered in a ward using multilevel linear regression analyses (*N* = 255, 17 wards).

	Model 2-1			Model 2-2			Model 3-1			Model 3-2		
					
	Coef.	SE	*p*	Coef.	SE	*p*	Coef.	SE	*p*	Coef.	SE	*p*
Intercept	3.19	0.22	<0.001	3.19	0.22	<0.001	3.20	0.22	<0.001	3.21	0.22	<0.001
**Individual characteristics**												
Sex (women)	−0.02	0.05	0.741	−0.02	0.05	0.678	−0.02	0.05	0.697	−0.03	0.05	0.524
**Professions (ref. nurse)**												
Doctor	0.32	0.13	0.016	0.32	0.13	0.017	0.32	0.13	0.015	0.32	0.13	0.016
Occupational therapist	0.20	0.10	0.054	0.21	0.10	0.044	0.20	0.10	0.056	0.21	0.10	0.046
Psychiatric social worker	−0.33	0.12	0.008	−0.34	0.12	0.006	−0.32	0.12	0.009	−0.34	0.12	0.005
Others	−0.01	0.11	0.901	0.00	0.11	0.970	−0.01	0.11	0.917	0.00	0.11	0.996
Years of experience	−0.02	0.05	0.741	0.00	0.00	0.700	0.00	0.00	0.617	0.00	0.00	0.733
Administrative position	0.01	0.10	0.909	0.01	0.10	0.955	0.01	0.10	0.946	−0.01	0.10	0.940
Employment status (full-time)	−0.05	0.21	0.818	−0.03	0.21	0.904	−0.05	0.21	0.798	−0.02	0.21	0.906
Experience of the committee for minimizing confinement	−0.03	0.07	0.621	−0.04	0.07	0.503	−0.03	0.06	0.642	−0.04	0.06	0.527
**Ward effects**												
Ward type (ref. ordinary or recuperation wards)												
Acute wards	−0.19	0.07	0.009	−0.18	0.07	0.006	−0.17	0.07	0.018	−0.07	0.08	0.333
Dementia wards	−0.01	0.12	0.909	−0.02	0.11	0.860	−0.02	0.11	0.871	−0.01	0.10	0.898
Number of seclusion rooms	0.01	0.02	0.572	0.01	0.01	0.663	0.02	0.02	0.299	0.00	0.01	0.744
Total time of seclusion per patients	0.00	0.00	0.849									
Total time of restraint per patients				0.00	0.00	0.187						
Total number of seclusion per patients							−0.41	0.48	0.391			
Total number of restraint per patients										−1.17	0.43	0.007

Coef., coefficient; SE, standard error; ref., reference; 95% CI, 95% confidence interval.

## Discussion

In this study, we developed the Japanese version of SACS and confirmed its reliability and validity. The total score of SACS had adequate internal consistency and test-retest reliability. Structural validity, being based on the original three-dimensional structure, was partially confirmed. Within-ward correlation of the score was confirmed, however hypothesized associations with the actual use of seclusion/restraints in a ward were not identified. Ceiling or floor effects were not observed.

Concerning structural validity, the result of the CFA based on the original three-dimensional structure revealed that, although its goodness-of-fit indices were marginally acceptable, the original subscale I (offending) did not explain the items included. The results of the EFA revealed a four-factor structure and showed that the original subscale I (offending) contained two factors, that is, one indicating the use of coercion as offending, and the other indicating it as avoidable or can be decreased to some extent. As for the original subscales II (care and security) and III (treatment), the EFA produced two factors corresponding to these two subscales, except for item 11. Item 11, which was originally categorized as an item of care and security, loaded on the factor of treatment in our EFA. However, it also loaded on the factor of care and security to a similar extent, and it seems acceptable to suppose it also belonged to the factor of care and security, as was the case in the original scale. Additionally, although items 5 and 6 loaded on two factors almost equally, their highest loading was consistent with the original subscale. In CFA and EFA, the structure corresponding to the original subscales II and III were moderately replicated, while the original subscale I was divided into two factors, which indicated the structural validity was partially confirmed. From the perspective of the therapeutic and safety paradigms presented by Doedens et al. (5), the original subscales III (treatment) and II (care and security) seemed to correspond to the dominant beliefs in the therapeutic and safety paradigms, respectively. They were almost replicated in our study. On the other hand, the original subscale I (offending) seemed to represent the undesirableness of the use of coercion contained in the safety paradigm. In our study, the belief that the use of coercion is avoidable was separated from those that it is harmful or undesirable. It was thought that a hope of decreasing the use of coercion was derived from the undesirableness of its use. Concerning the results of factor analyses conducted in the other language versions of the SACS, the original three-factor structure was almost replicated in the Polish version (18, 19), but not in the German version, which offered a one-factor structure ranging from rejecting to approving the use of coercion (20). Studies on the factor structure of the SACS is limited (17) and further studies are needed.

Concerning reliability, Cronbach’s alpha coefficient and ICC of the total SACS showed good internal consistency and test-retest reliability, respectively. Cronbach’s alpha coefficient in our study (0.761) was comparable to that of the original SACS (0.78) (16) and those of the other studies, which ranged from 0.58 to 0.84 (17). Cronbach’s alpha coefficient of the original subscale I was low, which was consistent with the above-mentioned results of EFA, suggesting two factors were included in the original subscale I. As for the test-retest reliability, the ICC of items 6, 13, and 15 were low, which describes the amount of coercion and the possibility of its reduction. These perceptions might have a tendency to fluctuate with the current state of the ward and seemed to be less stable across time. Based on these results, using the Japanese version of SACS as a total scale seemed acceptable, while using the original three subscales was not recommended.

The hypothesis that the score of SACS was correlated within wards was supported by the ward level variance and ICC calculated in the multilevel linear regression analysis. This suggested the scale’s construct validity, since the within-ward correlation was thought to stem from the respondents answering regarding the representative attitudes of their ward, as we required. On the other hand, the hypothesis of the positive association between the score of SACS and the actual use of seclusion/restraints was not supported. Previous studies also did not identify the associations between the score of SACS and the actual use of coercive measures (36, 37). Actual use of seclusion/restraints is known to be highly affected by patients’ characteristics, including symptoms, diagnosis, mental health problems, admission status, age, and sex (36, 38–45), as well as organizational factors such as the location of the institution, composition of staff, ward size, and ward design (36, 39, 44, 46, 47). These factors were not adjusted for in our study, which could be a reason for the null-association between the staff’s attitudes and the use of seclusion/restraints in our study.

In this study, we explored the association between the staffs’ attitudes toward the use of coercion and the actual use of seclusion/restraints based on the hypothesis that the staffs’ attitudes should directly affect their use of seclusion/restraints to some extent. We also expected that the negative attitudes of the staff toward the use of coercion may encourage the adoption of organizational efforts aimed at reducing the use of coercion in their institutions, such as a training program on alternative measures (12, 13), development of the detailed guidelines for the use of seclusions and restraints (48), and routine administration of the post-seclusion/restraint review (49–51). These organizational efforts are thought to not only promote reduced use of coercion in an institution but also affect its staffs’ attitudes [e.g., (12, 13)]. Future studies should explore these mutual interactions when examining the effects of the staffs’ attitudes toward the use of coercion.

As for item 4, “Use of coercion is a declaration of failure on the part of the mental health services,” it did not load on any factors in our EFA. It seemed to suggest a common problem observed in previous studies that developed the Polish version of SACS, which reported almost the same factor structure with the original version, except for item 4 (18, 19). The term “a declaration of failure” might be confusing or evoke different images among respondents. The response for item 4 seems to differ not only based on the attitude toward coercion but also on the attitude for the mental health services, and how to apprehend the failure of services in mental health fields. There were discussions among the authors on whether this item can be used in Japan from the translational stage. In Japan, coercive measures, such as seclusions and restraints, are routinely used based on the legislation, with their necessity and validity being properly examined. In such a circumstance, one could be perplexed or even feel blamed for his/her routine practice when seeing the term “a declaration of failure” abruptly, which might also affect his/her responses to the other items. For the Japanese version of SACS, it may be better to remove item 4. The Cronbach’s alpha coefficient of the 14 items without item 4 was 0.7575, and the same four-factor structure was replicated by EFA after removing item 4 (results not shown).

### Limitations

This study has several limitations. First, we conducted our survey in two psychiatric hospitals. The staff belonging to the same institution may share its culture, guidelines, and training, and may constantly relocate between wards, which could limit the diversity of attitudes toward coercion between wards. This may partly explain the significant but small variance between wards of the staff’s attitudes in our study, as compared to those found in a previous study (35). Second, in the 17 wards in which the study was conducted, there were two wards in which no seclusions were used and five wards in which no restraints were used during the 3-month study period. It was possible that 3 months was too short that the use of seclusion/restraints in a ward was heavily affected by the occasional characteristics of inpatients during the period, which might obscure the influence of the staff’s attitudes toward coercion. Third, when exploring the associations between the SACS score and the actual use of seclusion/restraints in a ward, we could not adjust for the patients’ characteristics such as diagnosis and clinical symptoms, which might affect the use of seclusion/restraints (36, 38–45). Fourth, we conducted our study in urban areas. A previous study reported that more seclusion/restraints were used in urban areas compared to rural areas (36). One possible reason is that patients admitted to hospitals in urban area are less known to the staff and more likely to be treated by coercive measures. It is possible that the reason to use coercion might vary between urban and rural areas, which may limit the generalizability of our results to rural areas. Fifth, the response rate of our survey (67.1%) was not high. It is possible that staff who were more interested in the use of coercion were more likely to respond to the survey, which might have caused selection bias. However, according to a systematic review of studies reporting the measurement properties of the SACS (17), the response rate of seven studies ranged from 13.8 to 91%, of which only two reported a rate higher than 70%. Therefore, the response rate of our study seemed acceptable considering similar surveys. Sixth, while the test-retest reliability of the total scale was acceptable, the sample size used to assess it was small. Furthermore, there were several items with low reliability; whether this suggested low test-retest reliability or a true change between the first and the second survey was unclear. Concerning test-retest reliability, further studies with a larger sample size and other indicators assessing the possibility of the true change in the wards are needed.

In future studies, exploring the associations of the SACS score with the actual use of seclusion/restraints are required with an adjustment for the patients’ characteristics among a wide variety of institutions, including those in rural areas. Furthermore, examining whether the SACS score changes by the interventions focusing on the use of coercion is required. The four-factor structure of the attitudes toward coercion suggested in our EFA also needs to be further explored. Particularly, exploration of the dimension which more strongly predicts the actual use of coercion, or that which are changeable by interventions, will proceed the understanding of the contribution of the attitudes toward coercion and efforts to reduce its use in Japan.

## Conclusion

We developed the Japanese version of SACS and confirmed its reliability and validity. In the Japanese version, the original three-dimensional structure was not replicated. Using the total score of SACS seemed reasonable in Japan.

## Data availability statement

The raw data supporting the conclusions of this article will be made available by the authors, without undue reservation.

## Ethics statement

The studies involving human participants were reviewed and approved by the Ethics Committee of the National Center of Neurology and Psychiatry, Japan. Written informed consent for participation was not required for this study in accordance with the national legislation and the institutional requirements.

## Author contributions

MF, MM, and TK developed the questionnaire. MM conducted the data collection. MF performed the analysis. MF wrote the first draft of the manuscript and all authors commented on previous versions of the manuscript. All authors contributed to the study conception and design, and read and approved the final manuscript.
