# Supplementary material for: Development of the Japanese version of Staff Attitude to Coercion Scale
Source: Front Psychiatry. 2022 Oct 17;13:1026676. doi: 10.3389/fpsyt.2022.1026676 (PMC9618617; doi:10.3389/fpsyt.2022.1026676)
Supplement: Supplementary file 1 [file Table_1.DOCX]

| Supplemental table 1. Ward information (N=17 wards) | | | | | |  |  |  |  |  |
| --- | --- | --- | --- | --- | --- | --- | --- | --- | --- | --- |
| Ward |  |  |  |  |  |  | Seclusion |  | Restraint |  |
| Institution | Ward ID | Ward type | Number of beds | Number of seclusion rooms | Number of nurses | Mean number of patients/day | Number of seclusions ^a)^ | Total time of seclusions (minutes)^b)^ | Number of restraints ^a)^ | Total time of restraints (minutes)^b)^ |
| A | 1 | Ordinary | 60 | 0 | 21 | 50.6 | 1 | 6552.5 | 3 | 4322.3 |
| A | 2 | Ordinary | 54 | 3 | 20 | 50.5 | 6 | 26427.1 | 1 | 15797.7 |
| A | 3 | Ordinary | 54 | 3 | 21 | 49.6 | 7 | 12555.5 | 3 | 6692.8 |
| A | 4 | Acute | 51 | 3 | 23 | 39.2 | 11 | 4466.1 | 10 | 8387.9 |
| A | 5 | Dementia | 51 | 0 | 16 | 50.8 | 0 | 0.0 | 2 | 3502.8 |
| B | 6 | Ordinary | 60 | 0 | 19.3 | 57.9 | 1 | 1370.7 | 0 | 0.0 |
| B | 7 | Recuperation | 60 | 0 | 20 | 59.2 | 0 | 0.0 | 0 | 0.0 |
| B | 8 | Ordinary | 60 | 4 | 19.7 | 60.0 | 10 | 7989.2 | 0 | 0.0 |
| B | 9 | Recuperation | 60 | 4 | 19.7 | 58.6 | 14 | 8294.0 | 0 | 0.0 |
| B | 10 | Ordinary | 60 | 4 | 21 | 57.8 | 8 | 2693.3 | 2 | 1451.1 |
| B | 11 | Ordinary | 60 | 4 | 19 | 57.5 | 9 | 6722.1 | 5 | 2671.2 |
| B | 12 | Ordinary | 60 | 0 | 19.5 | 53.2 | 2 | 687.8 | 1 | 255.6 |
| B | 13 | Ordinary | 46 | 6 | 20 | 43.8 | 11 | 3215.3 | 1 | 43.3 |
| B | 14 | Ordinary | 46 | 6 | 19 | 44.2 | 8 | 2757.5 | 1 | 9.2 |
| B | 15 | Acute | 39 | 6 | 21 | 36.2 | 12 | 3503.8 | 3 | 304.6 |
| B | 16 | Acute | 39 | 6 | 19 | 35.9 | 6 | 2758.0 | 2 | 657.9 |
| B | 17 | Acute | 50 | 2 | 21.2 | 44.1 | 1 | 337.4 | 0 | 0.0 |
| a) Total number of seclusion/restraints used during the previous three months in a ward | | | | | | | | | | |
| b) Total time of seclusion/restraints calculated by summing up all of them performed during the previous three months in a ward | | | | | | | | | | |
